# The Water-Alkane Interface at Various NaCl Salt Concentrations: A Molecular Dynamics Study of the Readily Available Force Fields

**DOI:** 10.1038/s41598-017-18633-y

**Published:** 2018-01-10

**Authors:** Thomas R. Underwood, H. Chris Greenwell

**Affiliations:** Department of Earth Sciences, Durham University, South Road, Durham, DH1 3LE United Kingdom

## Abstract

In this study, classical molecular dynamic simulations have been used to examine the molecular properties of the water-alkane interface at various NaCl salt concentrations (up to 3.0 mol/kg). A variety of different force field combinations have been compared against experimental surface/interfacial tension values for the water-vapour, decane-vapour and water-decane interfaces. Six different force fields for water (SPC, SPC/E, TIP3P, TIP3Pcharmm, TIP4P & TIP4P2005), and three further force fields for alkane (TraPPE-UA, CGenFF & OPLS) have been compared to experimental data. CGenFF, OPLS-AA and TraPPE-UA all accurately reproduce the interfacial properties of decane. The TIP4P2005 (four-point) water model is shown to be the most accurate water model for predicting the interfacial properties of water. The SPC/E water model is the best three-point parameterisation of water for this purpose. The CGenFF and TraPPE parameterisations of oil accurately reproduce the interfacial tension with water using either the TIP4P2005 or SPC/E water model. The salinity dependence on surface/interfacial tension is accurately captured using the Smith & Dang parameterisation of NaCl. We observe that the Smith & Dang model slightly overestimates the surface/interfacial tensions at higher salinities (>1.5 mol/kg). This is ascribed to an overestimation of the ion exclusion at the interface.

## Introduction

The liquid-liquid interface plays an important role in many physical, chemical and biological processes. From phase transfer catalysis to liquid chromatography; liquid-liquid extraction to pharmaceutical drug delivery^1^. In particular, the interface between water and oil is becoming increasingly examined within the scientific community. Recent work by Mugele *et al*. introduces an intriguing unsolved problem: that the wettability of a surface can be manipulated by altering the ionic composition of the surrounding fluid^2^. Mugele *et al*. examined the three-phase wettability of a mineral-oil-water system, measuring contact angle variations between mica, water and alkane as a function of salt concentration and composition. Despite extensive scientific efforts, the underlying mechanisms driving this wettability change are yet to be fully discerned, especially at the molecular level^3^. Mugele *et al*. proposed several models explaining the behaviour, and further challenged the computational chemistry community to help explain the phenomenon.

Classical MD simulations of salinity induced wettability alterations are becoming more prevalent in literature. Work by Jiménez-Ángeles & Firoozabadi^4^ attempt to solve the problem posed by Mugele directly. The study systematically measures the contact angle formed at the three-phase interface at varying salt concentrations. The dynamic properties of wettability as a function of salinity have further been examined by Zhang *et al*.^5^, who examine the non-equilibrium flow of oil through brines in various nanopores. No single force field (the input parameters for the MD simulation) is truly universal, and most MD studies of salinity induced wettability alterations combine at least three separate force fields without prior validation of their respective interfacial properties. Indeed, in the authors previous work^6,7^, the CGenFF^8–10^ model of oil was combined with the TIP3Pc^8^ water model and the ClayFF^11^ force field to model the mineral surface without thorough appreciation of the interfacial properties in the system. Without prior validation of the interfacial properties at the mineral-oil, mineral-water and water-oil interface, the subsequent results of such MD simulations can be questioned. Consequently, it is becoming increasingly important to understand the properties of the noted interfaces and to validate their respective properties against experimental data. In the present study, we focus on the water-oil interface, as it possesses a variety of interesting features, and has a large set of experimental data to compare against. Using classical MD simulations, we are able to examine the properties of the oil-water interface with atomic resolution, and how these properties change upon introduction (or depletion) of inorganic salts.

Experimental studies of the atomic structuring of fluid-fluid interfaces have historically been hindered by the lack of appropriate techniques capable of probing the interface with sufficient resolution. Recently, however, surface sensitive experimental techniques, such as sum frequency generation (SFG) spectroscopy, second harmonic generation (SHG) spectroscopy, and x-ray reflectivity measurements, have been able to probe the properties of fluid-vapour and fluid-fluid interfaces^12^. For example, Braslau *et al*. measured the surface width of the water-vapour interface (the distance between bulk-like liquid region of water and bulk-like vapour region) as 3.2 Å using x-ray reflectivity and capillary wave theory^13^, whilst similar experiments by Sanyal *et al*. discerned the ethanol-vapour interfacial width as 6.9 Å^14^. The interface between water and alkane has also been experimentally measured using x-ray reflectivity, as in the study of Mitrinovic *et al*., who measured the width of the water-hexane interface as 3.5 Å^15^ and Tikhonov *et al*., who measured the interfacial width of water-docosane (C _22_ H _46_) as 5.7 Å^16^.

Such advances in experimental techniques have been mirrored in the computational realm. Classical molecular dynamics (MD) simulations have been used to help interpret the phenomena present at the interfaces of water-vapour^17^, electrolyte-vapour^18^ and water-alkane^19,20^. The interfacial properties of water-vapour were calculated by Matsumoto & Kataoka in the late 1980s^17^, whilst the role of salts at the water-vapour interface was studied critically by Jungwirth & Tobias^18^. Water-alkane interfaces have also been heavily studied, for example by van Buuren *et al*., whom looked at the interface between water and decane^19^, and by Rivera *et al*., who looked at the water-alkane interface with the addition of methanol^20^. More recently, computation techniques have been able to resolve the *intrinsic* density profile of the liquid-vapour and liquid-liquid interface. That is, the interface between two phases excluding the contribution of thermal fluctuations (capillary waves), which act as to smear the density profiles across the interface. For example, Partay *et al*. were able to examine the intrinsic interface of the water-vapour system^21^, whilst Hantal *et al*. examined the interface between water and CCl_4_^22^. Using such techniques improves the clarity of the molecular structuring at the interface, and is seeing increasing use when studying the water-alkane interface^23,24^. The examination of the intrinsic density profile frequently leads to more insightful observations from the simulations.

As previously stated, no single force field is truly universal. Many different water models exist in the literature, and most excel at capturing certain aspects of water’s unusual physical properties. For example, the TIP4P2005 model excels at modelling the phase behaviour of water over a variety of temperatures and pressures, especially when compared to other models such as SPC/E^25,26^. However, the SPC/E model of water more accurately reproduces the experimental dielectric constant of water^25,26^. As no one water model is truly universal, it is not uncommon to observe various models being used in three-phase MD simulations. Commonly used water models applied to low-salinity EOR include the SPC^27^, SPC/E^28^, TIP3P^29^ and TIP4P^29^ parameterisations. Similar arguments also apply to the various parameterisation of oil molecules. Typical oil models used in previous work include CGenFF^8^, TraPPE^30^ and OPLS^31^. Both TraPPE and OPLS were parameterised to model organic solvents (with TraPPE specifically parameterised to model alkanes)^30,31^. OPLS, however, is frequently combined with ClayFF^5,32^ (a force field frequently used to model mineral surfaces), despite the fact that both force fields are parameterised with different Lennard-Jones mixing rules, and are therefore not inherently compatible. In contrast to TraPPE and OPLS, CGenFF is more general. CGenFF offers fully automated atom typing, and therefore offers large throughput.

In the present work, we investigate the utility of various force fields to accurately reproduce the interfacial behaviour between water and alkane at different NaCl concentrations. We have used decane as a representative model alkane. The aim of the article is to interpret the phenomena of the liquid-liquid interface at the molecular level, and to trial various force fields for future use in more complex three-phase simulations. Classical MD simulations have been used to calculate the surface tension of water and decane in their liquid-vapour phases. The interfacial tension of the water-decane interface has also been examined. The properties of six different force fields for water (SPC, SPC/E, TIP3P, TIP3Pc, TIP4P and TIP4P2005), and three further force fields for alkane (CGenFF, OPLS-AA and TraPPE-UA) are compared against experimental data. The salinity dependence on surface/interfacial tension has been modelled using the Smith & Dang parameterisation of NaCl. Both the intrinsic and non-intrinsic density profiles for each interface has been calculated to show the structural similarities and differences between each force field.

## Results

### The Alkane-Vapour Interface

**Figure 1:**
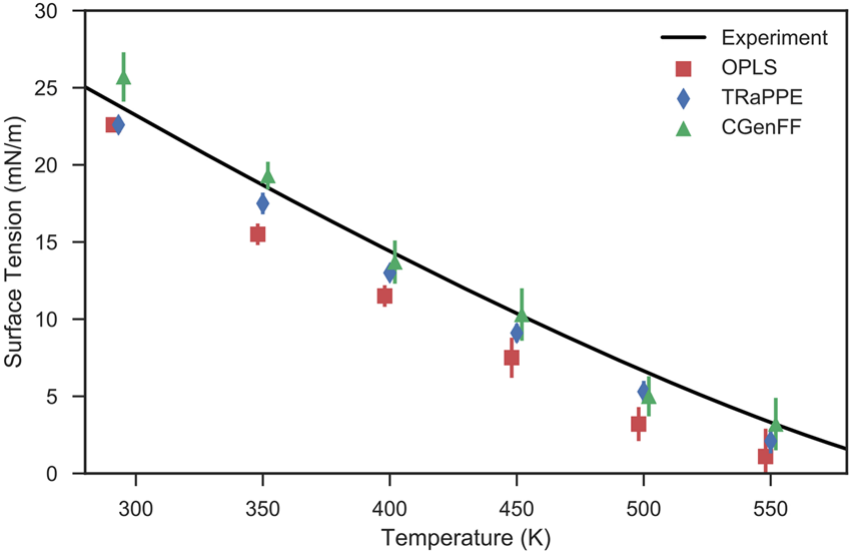
Surface tension of decane as a function of temperature. Data points have been offset for clarity. Experimental curve has been reproduced from the NIST standard reference database^51^ and from Jesper & Kring^58^. Error bars represent two standard errors of the mean above and below calculated surface tension values.

Figure 1 presents the calculated surface tension values of decane over a range of temperatures. The results show that the three tested force fields accurately reproduce the overall trend of surface tension at various temperatures.

Whilst we have examined decane solely, the considered force fields are widely transferable. Both OPLS-AA and TraPPE-UA have previously been shown to accurately predict the surface tension values of ethane through to hexadecane^33,34^. To the author’s knowledge, there have been no surface tension calculations of alkanes using the CGenFF force field.

Both OPLS-AA and TraPPE-UA systematically underestimate the surface tension of decane at all temperatures, OPLS more-so than TraPPE-UA. CGenFF overestimates surface tension at lower temperatures, and underestimates surface tension at higher temperatures. Our results concur with the work of Ismail *et al*., who show that OPLS-AA consistently underestimates the surface tension of various saturated *n*-alkanes^33^ and with Mendoza *et al*., who present that TraPPE-UA is generally in good agreement with experimental data^34^. Our results are also consistent with the study of Caleman *et al*., who show that OPLS-AA often underestimates the surface tension values of a wide variety of organic liquids^35^.

Previous work by Nicolas & Smit has shown that the accurate calculation of surface tension for saturated alkanes is linearly proportional to the liquid density predicted in the simulations^36^. Values for the liquid and vapour density of decane have been calculated by fitting the interfacial density profile to a **tanh** function (see Equation 6 in Methods). The liquid density (*ρ*_*l*_) of decane at 293.15 K is presented in Table 1, along with values for the interfacial width (Δ). Note that the vapour density (*ρ*_*v*_) at 293.15 K is calculated to be zero, and is therefore not presented.

**Table 1:** The surface tensions, liquid densities and interfacial widths of the decane-vapour interface at 293.15 K using various force fields. Two standard errors of the mean are presented in parentheses. Experimental datum has been reproduced from the NIST standard reference database^51^.

Oil Model	*γ*	${{\boldsymbol{\delta }}}_{{\boldsymbol{e}}{\boldsymbol{x}}{\boldsymbol{p}}}^{{\boldsymbol{s}}{\boldsymbol{i}}{\boldsymbol{m}}}$	*ρ*_*l*_	Δ
	(mN/m)	(%)	(g/cm^3^)	(Å)
TraPPE-UA	22.6 (0.4)	−5	729.8	6.0
CGenFF	25.7 (1.6)	+ 8	741.5	5.6
OPLS-AA	22.6 (0.4)	−5	730.3	5.9
Experiment^51^	23.823		730.33	

Our results show that both TraPPE-UA and OPLS-AA marginally underestimate the liquid density of decane at 293.15 K, and consequently, both underestimate the surface tension. Conversely, CGenFF overestimates the liquid density, and therefore the surface tension of decane. The accurate reproduction of liquid-vapour densities depends on the treatment of long-range dispersion forces^37^. Ismail *et al*. show that Lennard-Jones cutoffs greater than 16 Å are required to yield results in agreement with explicit long range methods (evaluated in reciprocal space)^33^, whilst the simulations of Mendoza *et al*. and Lopez *et al*. also show the utility of using Ewald-summations to treat these long range dispersion forces^34,38^. Note that our simulations satisfy both these criteria.

**Figure 2:**
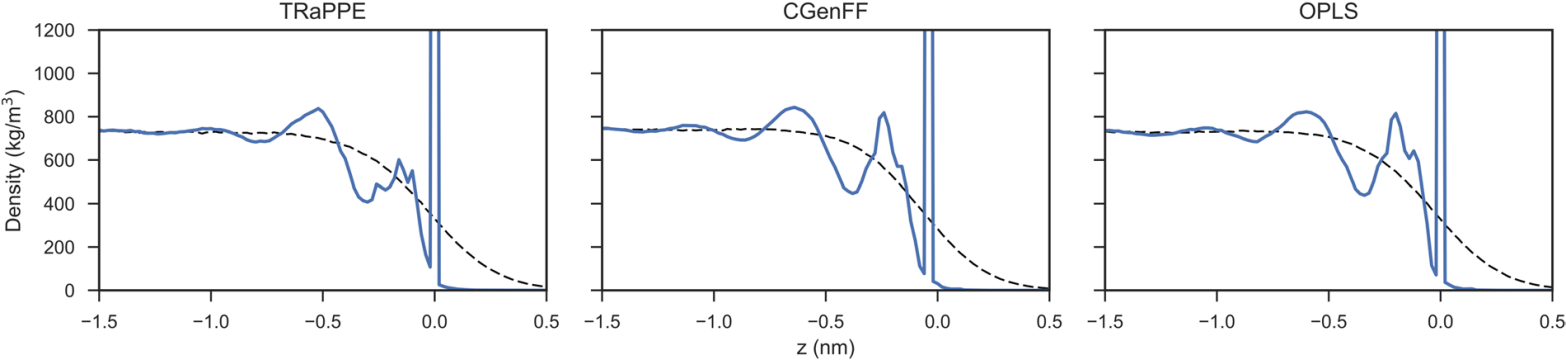
The intrinsic (bold line) and non-intrinsic (dashed line) interfacial density profiles of decane using various force fields. The zero point of the interface (z = 0 nm) corresponds to the position of outermost carbon atoms in the intrinsic density profile and the point of zero surface excess (the Gibbs dividing surface) of decane for the non-intrinsic density profile.

Figure 2 presents the interfacial density profiles (both intrinsic and non-intrinsic) of the decane-vapour interface at 293.15 K. The density profiles show that all three force fields act remarkably similarly, even though CGenFF and OPLS-AA are all-atom representations of decane, and TraPPE-UA is a united-atom representation. The intrinsic density profiles present four discrete peaks of decane before the density becomes bulk-like.

### The Water-Vapour Interface

The calculated surface tension values of pure water are presented in Table 2. It can be seen that almost all water models underestimate the experimentally observed surface tension of water by at least 15%. The only exception to this is TIP4P2005, which underestimates the surface tension of water by 7%. Our results compare favourably to the study of Vega & De Miguel^25^. Like their work, we find that the accurate reproduction of surface tension follows the trend:

**Table 2:** The surface tensions, liquid densities and interfacial widths of water-vapour interfaces using various force fields. Two standard errors of the mean are presented in parentheses. Experimental data is taken from the CRC Handbook of Chemistry & Physics^53^ and Braslau *et al*.^13^.

Water Model	*γ*	${{\boldsymbol{\delta }}}_{{\boldsymbol{e}}{\boldsymbol{x}}{\boldsymbol{p}}}^{{\boldsymbol{s}}{\boldsymbol{i}}{\boldsymbol{m}}}$	*ρ*_*l*_	Δ
	(mN/m)	(%)	(g/cm^3^)	(Å)
SPC	54.1 (0.8)	−26	979.2	3.7
SPC/E	61.8 (0.7)	−15	1000.4	3.3
TIP3P	50.8 (0.6)	−30	987.8	3.9
TIP3Pc	55.8 (0.5)	−23	1016.9	3.7
TIP4P	56.3 (0.5)	−23	995.2	3.6
TIP4P2005	67.9 (0.7)	−7	997.2	3.2
Experiment^53^	72.8		998.2	3.2^13^

TIP4P2005 > SPC/E > TIP4P > SPC > TIP3P

The TIP3Pc model, which introduces LJ sites on hydrogen atoms, performs better than the original TIP3P parameterisation, but still underestimates the surface tension of water by 23%. We observe that, generally, the four-point water models perform better than the three-point models, in keeping with the observations of Vega & De Miguel^25^. Of all the three-point water models, SPC/E most accurately reproduces the experimental surface tension of water, whilst of the four-point water models, the TIP4P2005 parameterisation performs the best.

**Figure 3:**
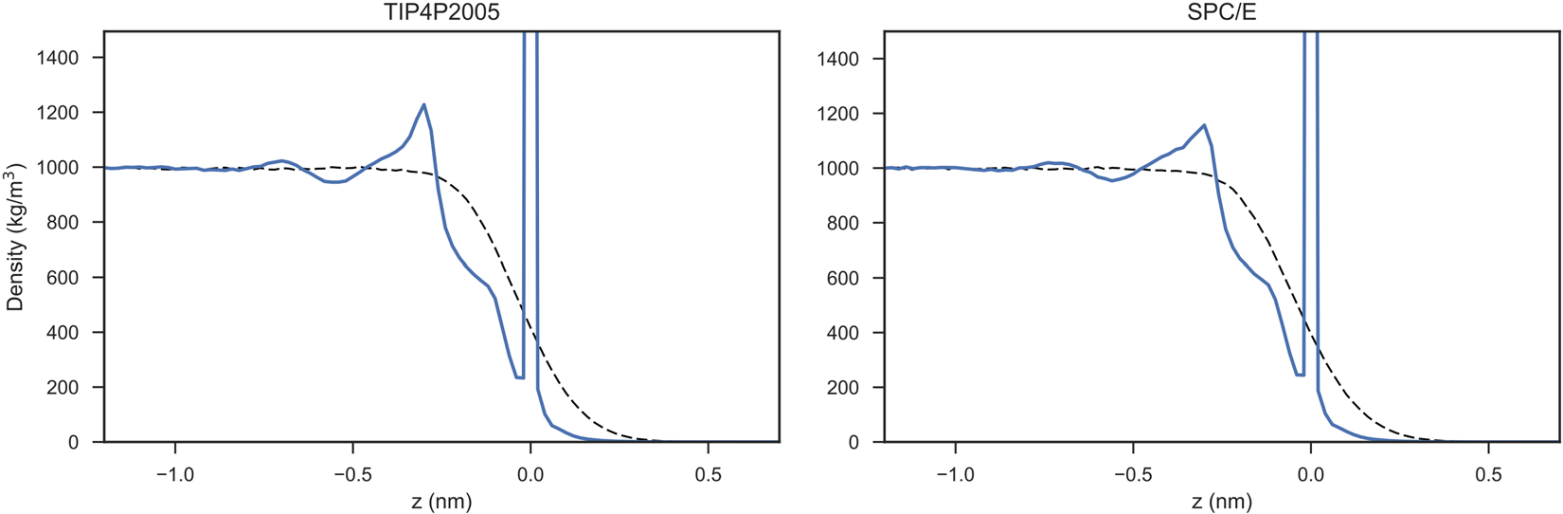
The intrinsic (bold line) and non-intrinsic (dotted line) density profile of TIP4P2005 and SPC/E water models. The zero point of the interface (z = 0 nm) corresponds to the position of outermost water atoms in the intrinsic density profile and the point of zero surface excess (the Gibbs dividing surface) of water for the non-intrinsic density profile.

The density profiles of TIP4P2005 and SPC/E water are presented in Fig. 3. Analysing the intrinsic density profiles, it can be seen that water forms three separate density peaks at the water-vapour interface. This information is lost due to the capillary wave fluctuations present in the simulation when examining the non-intrinsic density profile solely. Also notable, is that most water models appear to overestimate the interfacial width of water compared to the results of Braslau *et al*.^13^, see Table 2. The TIP4P2005 and SPC/E water model most accurately predict the experimentally observed interfacial width of water. Overall, the TIP4P2005 is best placed to model water-vapour interfaces. Of the three-point water models, SPC/E is the most favourable.

### The Water-Vapour Interface at Various NaCl Concentrations

**Figure 4:**
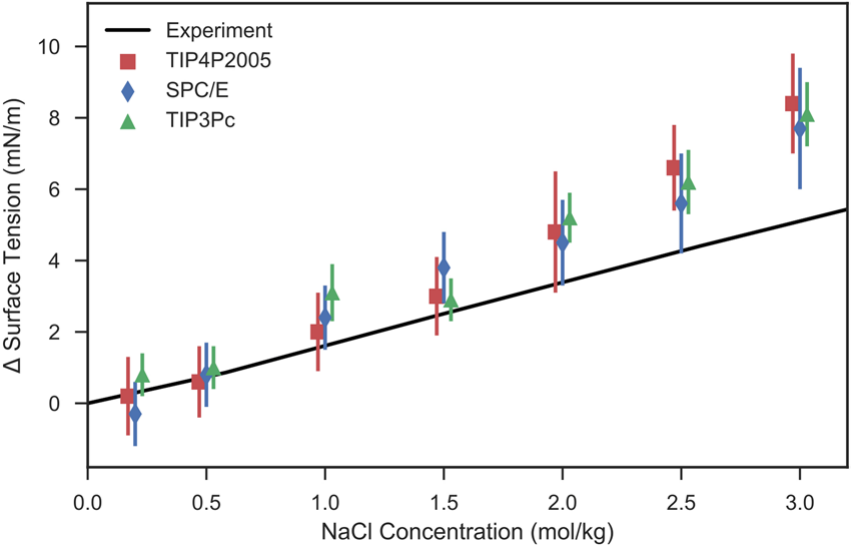
The variation of water-vapour surface tension with additional NaCl salts. The experimental curve is extrapolated using data from Vargaftik *et al*.^59^, Ali *et al*.^60^, and Aveyard & Saleem^42^.

Proceeding, we have chosen three water models to examine the trends in surface tension as a function of salinity. The TIP4P2005 and SPC/E water models were examined as they represent the best four-point and three-point water models respectively. The TIP3Pc water model was also examined, as it is frequently simulated in conjunction with secondary organic phases^6,7^. The variation of surface tension with NaCl concentration is presented in Fig. 4.

Firstly, we note that the overall trend of surface tension variation with NaCl concentration is well captured by the classical MD simulations. The simulations are able to accurately predict the experimental surface tensions of NaCl solutions up to 1.5 mol/kg, however, the simulations slightly overestimate the surface tension of NaCl electrolyte at higher concentrations, deviating more drastically as concentrations increase beyond 2.0 mol/kg. Similar behaviour was noted by D’Auria & Tobias, who observed that the surface tension of the TIP3P water model was overestimated when used in conjunction with the Smith & Dang parameterisation at NaCl concentrations of 1.2 mol/kg and 6.17 mol/kg^39^.

Secondly, we observe that the simulated surface tensions of all three water models agree with each other at each concentration. This implies that the accurate modelling of interfaces containing electrolytes depends more so on the parameterisation of the ion, rather than the water model. This conclusion concurs with the work of Neyt *et al*., who presented that the salinity dependence of the electrolyte-vapour interface varied primarily with ion parameterisation, rather than the water model, whilst the utilised water model sets the accuracy of the surface tension calculation without additional NaCl^40^. Notably, Neyt *et al*. presented that the TIP4P2005 water model used in conjunction with the OPLS parameterisation of NaCl, typically returned surface tensions within 0.2% of the experimental trend^40^.

### The Water-Alkane Interface at Various NaCl Concentrations

Table 3 presents the interfacial tension values of the decane-water interface, without salts, for various force field combinations as predicted by the MD simulations. We have chiefly examined TraPPE-UA and CGenFF due to their compatibility (*i*.*e*. consistent use of Lennard-Jones mixing rules) with ClayFF; which makes these force fields more suitable for use in complex three-phase simulations. Priority has also been placed on testing the TIP4P2005 and SPC/E water models, as these most accurately model the interfacial properties of pure water. TIP3Pc has also been examined as it is the default three-point water model used in conjunction with CGenFF^8^.

**Table 3:** The decane-water interfacial tensions calculated using a combination of various force fields. Two standard errors of the mean are presented in parentheses. Experimental values are averaged from Zeppieri *et al*.^61^, Goebel & Lunkenheimer^62^ and Aveyard & Haydon^63^.

Oil Model	Water Model	*γ*(mN/m)	${{\boldsymbol{\delta }}}_{{\boldsymbol{e}}{\boldsymbol{x}}{\boldsymbol{p}}}^{{\boldsymbol{s}}{\boldsymbol{i}}{\boldsymbol{m}}}$(%)
TraPPE-UA	TIP4P2005	56.7 (1.7)	+ 8
TraPPE-UA	TIP4P2005*	54.2 (1.0)	+ 3
CGenFF	SPC/E	55.3 (1.2)	+ 5
CGenFF	TIP3Pc	48.2 (1.2)	$-8$
CGenFF	TIP4P	51.9 (1.8)	$-1$
Experiment		52.5 (0.6)	

In addition to the usual TIP4P2005 water model interacting with TraPPE-UA, we have examined the parameters as described by Ashbaugh *et al*.^41^, denoted TIP4P2005*. The TIP4P2005* water model uses custom Lennard-Jones interactions between the oxygen atoms of water and the TraPPE-UA beads. These custom interactions have been finely tuned to accurately reproduce the hydration free energy of alkanes^41^. All other parameters of the TIP4P2005* water model are identical to the original TIP4P2005 parameterisation.

We note that the MD simulations are able to predict the interfacial tension of the water-decane interface within 10% of the empirically observed values. The simulations of this interface are notably more accurate than the simulations of the water-vapour interface. The combination of different force fields appears more robust for the water-decane interface, compared to the water-vapour interface. This is likely due to the absence of a vapour phase in the simulations, which when inaccurately modelled (in particular, the vapour density), can lead to deviations in the predicted surface tension, as noted by Nicolas & Smit^36^.

**Figure 5:**
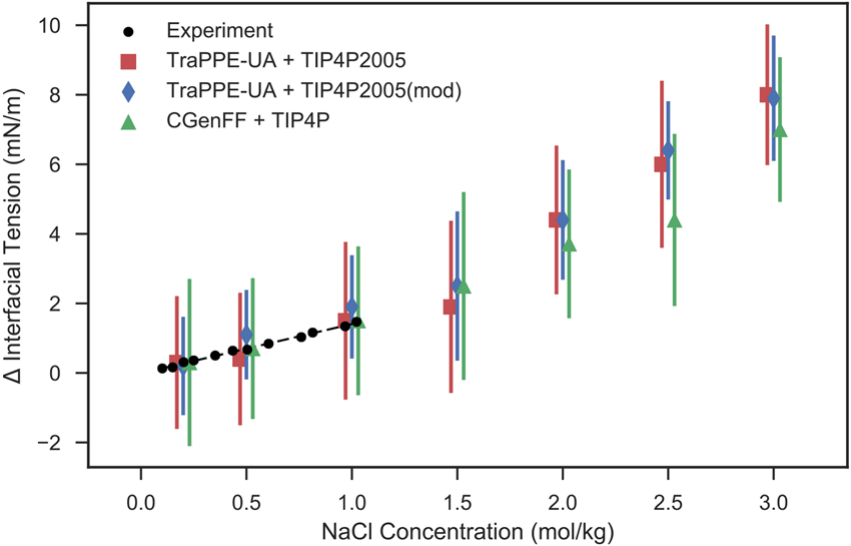
The variation of decane-water interfacial tension with additional NaCl salts. Experimental data are taken from Aveyard & Saleem^42^.

Figure 5 presents the deviation of decane-water interfacial tension with increasing NaCl concentration for various combinations of force fields. The computed interfacial tensions accurately follow the experimental trend observed by Aveyard & Saleem^42^ up to 1.0 mol/L. Much like the water-vapour interface, the different parameter sets agree with each other at each NaCl concentration. This again highlights that the ion parameterisation may be more important for the accurate modelling of the water-vapour/water-decane interface.

## Discussion

The presented results show the quality with which MD simulations can capture the bulk properties and structural properties of the liquid-liquid interface. One key advantage of such simulations, compared to many experiments, is the unparalleled resolution with which one can interpret the bulk phenomena on display. In this instance, we can use the MD simulations to further our understanding of *why* the surface tension at the water-vapour/water-alkane interface increases with increasing NaCl concentration. Typically, this behaviour is explained due to an exclusion of ions from the liquid-vapour interface^3^. This directly alters the surface tension between the two phases as described by the Gibbs adsorption isotherm:

1 $$d\gamma =-{{\rm{\Gamma }}}_{{\rm{Na}}}d{\mu }_{{\rm{Na}}}-{{\rm{\Gamma }}}_{{\rm{Cl}}}d{\mu }_{{\rm{Cl}}}$$

where Γ_*i*_ is the surface excess and *μ*_*i*_ is the chemical potential of species *i*∈{ Na, Cl }. In this study, the surface excess of Na^+^ and Cl^−^ ions across the electrolyte-water interface has been calculated as:

2 $${{\rm{\Gamma }}}_{i}={\int }_{-\infty }^{{z}_{{\rm{Gibbs}}}}[{\rho }_{i}(z)-{\rho }_{i}^{{\rm{liq}}}]dz+{\int }_{{z}_{{\rm{Gibbs}}}}^{\infty }[{\rho }_{i}(z)-{\rho }_{i}^{{\rm{vap}}}]dz$$

where *ρ*_*i*_(*z*) is the non-intrinsic density profile of phase *i* across the interface, ${\rho }_{i}^{{\rm{liq}}}$ is the bulk liquid density of phase *i*, and ${\rho }_{i}^{{\rm{vap}}}$ is the bulk vapour density of phase *i* (typically ${\rho }_{i}^{{\rm{vap}}}=0$ for ions in liquid-vapour simulations). *Z*_Gibbs_ is the location of the Gibbs dividing plane, which is priorly calculated by minimising Γ_water_, such that:

3 $${{\rm{\Gamma }}}_{{\rm{water}}}={\int }_{-\infty }^{{z}_{{\rm{Gibbs}}}}[{\rho }_{{\rm{water}}}(z)-{\rho }_{{\rm{water}}}^{{\rm{liq}}}]dz+{\int }_{{z}_{{\rm{Gibbs}}}}^{\infty }[{\rho }_{{\rm{water}}}(z)-{\rho }_{{\rm{water}}}^{{\rm{vap}}}]dz=0$$

**Figure 6:**
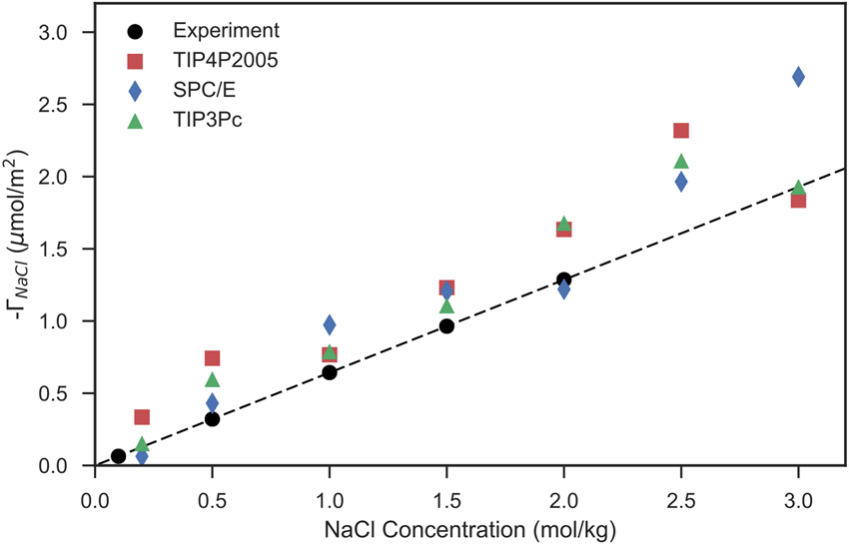
The surface excess of NaCl at the water-vapour interface as a function of NaCl concentration. Experimental data is taken from Ali *et al*.^43^.

Note that equation 3 has been used to centre the non-intrinsic density profiles presented in the results section. Figure 6 presents the negative total surface excess of ions (${{\rm{\Gamma }}}_{{\rm{NaCl}}}={{\rm{\Gamma }}}_{{\rm{Na}}}+{{\rm{\Gamma }}}_{{\rm{Cl}}}$) at the water-vapour interface calculated from all simulations as a function of NaCl concentration. We observe that, compared to the experimental data of Ali *et al*., the simulations marginally overestimate the negative total surface excess of NaCl, $-{{\rm{\Gamma }}}_{{\rm{NaCl}}}$, especially at higher concentrations^43^. In turn, this elucidates why the surface tension values are overestimated in the simulations at higher salt concentrations. The surface tension values are overestimated due to the overestimation of ionic exclusion at the liquid-vapour interface. Notably, the lack of polarization may explain why our classical molecular dynamics simulations overestimate the ionic exclusion. It is well known that, whilst ion exclusion generally increases with salt concentration, certain anions (for example, bromide and iodide) can accumulate at the interface due to the polarization^44^. Recent computational work by Neyt *et al*. and Neyt *et al*. has examined the effects of polarization on electrolyte-vapour interfaces^40,44^. Both studies concluded that current models of polarizable force fields (including both polarizable water and/or ions), are not mature enough to capture the salinity dependence on surface tension. These studies tested both the Drude oscillator model and the electronic continuum correction model (whereby classical point charges are shifted by a screening factor to account for polarization). Work by Jiang & Panagiotopolous further show that theelectronic continuum correction model fails to accurately predict the interfacial properties of electrolytes with polarizable models^45^. More recently, work by Jiang *et al*. presents that the BK3 polarizable model is able to accurately capture the interfacial properties of NaCl electrolyte solutions^46^. However, it is worth noting that such polarizable models typically run 5–10 times slower compared to the classical parameterizations used in this study, and that the implementation of the BK3 model currently only runs on a single processor using GROMACS (the software suite used in this study).

**Figure 7:**
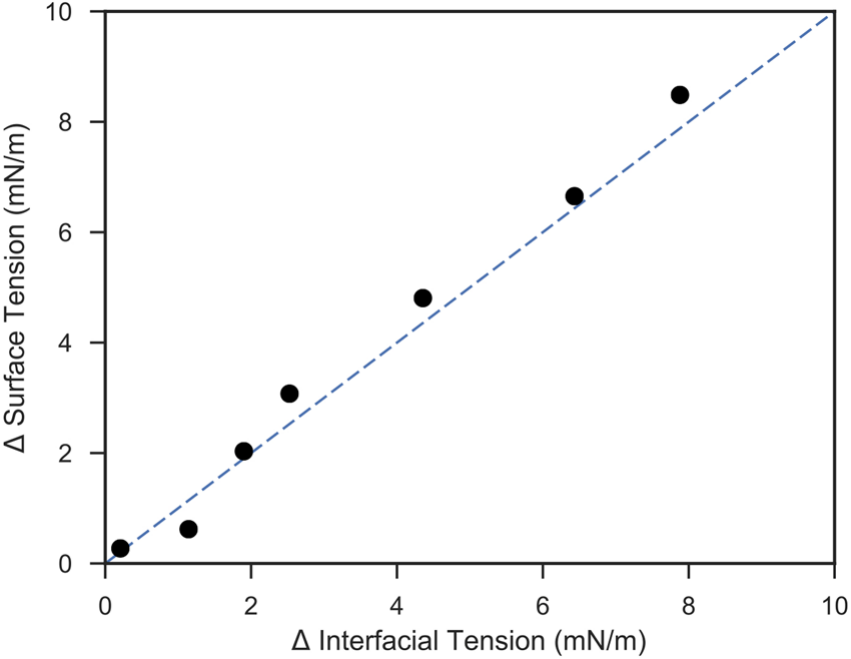
The change in surface tension at the water-vapour interface (y-axis) against the change in interfacial tension at the water-alkane interface (x-axis). The diagonal line represents when the two properties are linearly proportional.

Also noteworthy is that the variation in interfacial tension predicted at the interface between water and decane is remarkably similar to that at the water-vapour interface. This is apparent in Fig. 7, which compares the change in surface tension at the water-vapour interface against the change in interfacial tension at the water-alkane interface (using the TIP4P2005 water model and the TraPPE-UA oil model). The overall change in interfacial tension is therefore due to the properties primarily within the water phase. This result is somewhat unsurprising as the dielectric properties of decane and water vapour are much similar when compared to bulk liquid water. The surface tensions and interfacial tensions scale linearly as the ions are excluded from their respected interfaces in equal value, and the ions are not miscible in the vapour/decane phase respectively.

To conclude; in this study, the interfacial properties of water and decane have been examined at various NaCl concentrations using classical molecular dynamics simulations. By choosing an appropriate set of interaction parameters (force fields), one can obtain remarkable agreement between model and experimental observation. In particular, the TIP4P2005 water model is best placed to examine the interfacial properties of water. The SPC/E parameterisation is the best three-point water model to interpret the interfacial behaviour of water. CGenFF, OPLS-AA and TraPPE-UA all accurately reproduce the interfacial properties of decane. In combination, CGenFF and TraPPE-UA are compatible with various water models, and are able to accurately predict the interfacial tension of the water-decane interface. The salinity dependence on surface/interfacial tension is well captured using the Smith & Dang parameterisation of NaCl. We observe that the model slightly overestimates the surface/interfacial tensions at higher salinities. This is due to an overestimation of the ion exclusion at the interface. The simulations further suggest that the salinity dependence on surface/interfacial tension is dictated by the parameterisation of the salt ions. Future work will examine this hypothesis, and will model different ion parameterizations. Future work will also examine the role of divalent cations at the water-vapour/water-alkane interface, whereby, charge inversion may play a determining role on the behaviour of the liquid-liquid interface.

## Methods

The datasets generated and analysed during the current study are available from the corresponding authors on reasonable request.

All simulations were calculated using GROMACS 5.1.4^47^ with an electrostatic and van der Waals cutoff radii of 2.0 nm. Long range electrostatics were calculated using a Particle-Mesh-Ewald (PME) summation with grid spacings of 0.1 nm. The PME summation used a spline interpolation order of 4, and long-range electrostatic interactions were accurate to within 99.999%.

All simulations were initialised with an energy minimisation calculation to minimise any unphysical atomic overlaps. This was achieved using a steepest descents algorithm, which was terminated once the maximum force on any one atom was less than 100 kJ/(mol nm).

All simulations were subsequently equilibrated for 1 ns. Simulations of liquid-vapour phase surface tensions were calculated in the canonical ensemble (constant particle number - *N*, constant volume - *V* and constant temperature *T*) at 293.15 K, using a V-rescale thermostat set to rescale system temperatures every 0.5 ps. Liquid-liquid interfacial tension simulations were calculated in the isothermal-isobaric ensemble (constant particle number - *N*, constant volume - *V* and constant pressure - *P*) at 293.15 K and 1.01325 bar. *NPT* simulations were equilibrated using a V-rescale thermostat with a temperature coupling constant of 0.5 ps. Pressure coupling was achieved using a Berendsen barostat, with a pressure coupling constant of 1 ps.

Following equilibration, all simulations were run for a production period of 10 ns. During the production period, all simulations used a Nosé-Hoover thermostat with a temperature coupling constant of 0.5 ps and a Nosé-Hoover chain length of 1. *NPT* simulations used an isotropic Parrinello-Rahman barostat during the production period, with a pressure coupling constant of 1 ps. Unless otherwise stated, all production simulations have been calculated at 293.15 K and 1.01325 bar.

Three different force field parameterisations for decane have been tested in this study: TraPPE-UA^30^, OPLS-AA^31,48^ and CGenFF^8–10^. TraPPE-UA is a united atom force field, where CH_3_- and -CH_2_- groups are modelled as soft spheres. OPLS-AA and CGenFF are both all atom models, where carbon and hydrogen atoms are modelled explicitly. Six different water models were examined in the water-vapour interface: SPC^27^, SPC/E^28^, TIP3P^29^, TIP3Pc^8^, TIP4P^29^ and TIP4P2005^49^. TIP3Pc refers to the CHARMM variant of the original TIP3P parameterisation, whereby hydrogen atoms contain Lennard-Jones sites. The Smith and Dang parameterisation has been used to model aqueous sodium and chloride ions^50^.

### System Setup

Three different systems are presented in this study. In the first, a 5 × 5 × 5 nm^3^ box of 392 decane molecules is inserted into 5 × 5 × 20 nm^3^ simulation box. In the second, a 5× 5× 5 nm^3^ box of 4139 water molecules is inserted into 5 × 5× 20 nm^3^ simulation box. The number of decane and water molecules in each film is calculated to match the bulk density of each solvent at 293.15 K and 1.01325 bar, 0.727 g/cm^3^ for decane and 0.9982 g/cm^3^ for water respectively^51^. In the third system, the decane and water films are combined in a 5 × 5× 10 nm^3^ simulation box. Each system was generated using the Packmol software package^52^. Systems involving water were further examined at various NaCl concentrations. Systems were setup in terms of NaCl molal concentration, up to a maximum of 3.00 mol/kg, in increments of 0.50 mol/kg. This was achieved by replacing water molecules with the relevant amount of sodium and chloride ions. The number of water molecules and ions present in each simulation is presented in Table 4. Additionally, 0.20 mol/kg NaCl solution was examined. Whilst simulation results are presented in molal concentration (mol/kg), experimental results are often presented in terms of molar concentration (mol/L). The conversion from molar concentration to molal concentration is presented in Table 4, using data extrapolated from the CRC Handbook of Chemistry & Physics^53^.

**Table 4:** The amount of water molecules, aqueous sodium ions and aqueous chloride ions present in each simulation. Conversions between molal and molar concentrations have been calculated using the density of NaCl electrolyte at various concentrations^53^.

*m*(mol/kg)	N_water_	N_Na_	N_CI_	Mass_NaCI_(%)	*ρ*(g/cm^3^)	M(mol/L)
0.00	4139	0	0	0.0	0.998	0.00
0.20	4109	15	15	1.1	1.005	0.20
0.50	4063	38	38	2.9	1.018	0.51
0.00	3989	75	75	5.7	1.040	1.02
0.50	3913	113	113	8.6	1.061	1.56
0.00	3837	151	151	11.3	1.082	2.10
0.50	3763	188	118	13.9	1.102	2.63
0.00	3687	226	226	16.6	1.122	3.17

### Analyses

Thermodynamic data from each simulation were output every 1 ps. Final values for thermodynamic quantities were averaged over all 10 ns, and errors were calculated using a block-averaging method, with each block averaging over a 1 ns timeframe. In all figures, error bars are presented to ±2 standard errors of the mean (a confidence interval of 95%).

The surface tension across an interface has been calculated using the diagonal components of the local pressure tensor:

4 $$\gamma =\frac{1}{2}{\int }_{0}^{{L}_{z}}[{p}_{{\rm{N}}}(z)-{p}_{{\rm{T}}}(z)]dz$$

where *L*_z_ is the length of the simulation in *z* (the direction normal to the interface) and *p*_N_(*z*) & *p*_T_(z) represent the normal and tangential components of the pressure tensor with respect to the interface:

5 $${p}_{{\rm{N}}}(z)=\frac{{p}_{{\rm{xx}}}(z)+{p}_{{\rm{yy}}}(z)}{2}\quad \quad \quad {p}_{{\rm{T}}}(z)={p}_{{\rm{zz}}}(z)$$

The diagonal components of the local pressure tensor (*p*_xx_, *p*_yy_ & *p*_zz_) have been calculated using the Irving-Kirkwood formalism^54^.

Density profiles across the interface have been calculated following a two-stage process. Firstly, the simulation trajectory is centred about the centre of mass of the primary solvent phase in each simulation (typically water). This reduces artefacts caused by the collective drift of the interface throughout the simulation. The primary phase is calculated by clustering all molecules in the system. The largest cluster is then selected as the primary (liquid) phase. Any molecule further than 0.35 nm from the primary phase is excluded from the centre of mass calculation, and therefore does not affect the centering of the system. A cutoff of 0.35 nm was selected as this corresponds to the first minimum in the radial distribution function (RDF) of water^55,56^. Consequently, water molecules in the vapour phase are excluded in the centre of mass calculation for the bulk liquid water phase, and therefore do not artificially shift the resulting density profiles normal to the interface. After the system has been centred, the density profile is calculated across the interface using a bin size of 0.01 nm. Where applicable, the density profile has been fit to the equation:

6 $$\rho (z)=\frac{{\rho }_{l}+{\rho }_{v}}{2}+\frac{{\rho }_{l}-{\rho }_{v}}{2}\,\tanh (\frac{z-{z}_{0}}{\sqrt{2}{\rm{\Delta }}})$$

where ${\rho }_{l}$ is the liquid density of the primary phase, ${\rho }_{v}$ is the vapour density of the primary phase, z_0_ is the location of the interface, and Δ is the interfacial width. The density profiles of Na^+^ and Cl^−^ ions have subsequently been calculated relative to the definition of the water interface.

The density profiles calculated using the above methodology are subject to capillary waves due to thermal fluctuations. Recently, computation techniques have been able to resolve the *intrinsic* density profile of the liquid-vapour and liquid-liquid interface^21^. That is, the interface between two phases excluding the contribution of thermal fluctuations (capillary waves), which act as to smear the density profiles across the interface. The amplitude of these capillary waves scales as:

7 $$\langle {\xi }^{2}(q)\rangle \propto \frac{kT}{q}$$

where the maximum wave vector, *q*, depends upon the size of the simulated interface.

The calculation of the intrinsic density profile has been evaluated by offsetting the amplitude of the thermal fluctuations (ξ) from the interface:

8 $${\rho }_{i}(z)=\frac{1}{A}\langle \sum _{i}\delta (z-{z}_{i}+\xi ({x}_{i},{y}_{i}))\rangle $$

where index *i* sums over all atoms of phase *i*, and *z* is the position of the local non-intrinsic interface. The intrinsic density profiles have been calculated using the ITIM method^21^ as presented by Sega *et al*.^57^, using a probe sphere radius of 0.2 Å. Within the ITIM method a probe sphere is moved along test lines perpendicular to the plane of the fluid-vapour or fluid-fluid interface. Once the probe sphere touches the first atom within of the phase of interest, this molecule is marked as being interfacial. This process is repeated over the entire interfacial area in the simulation. The intrinsic density profile is then calculated using the offset (*ξ*) of the marked interfacial atoms.
